# An analytic observational study on complaints management in the general practice out of hours care setting: who complains, why, and what can we do about it?

**DOI:** 10.1186/s12875-016-0484-1

**Published:** 2016-07-21

**Authors:** Ruth A. Barragry, Leo E. Varadkar, David K. Hanlon, Ken F. Bailey, Tom C. O’Dowd, Brendan J. O’Shea

**Affiliations:** TCD HSE GP Training Scheme, Tallaght Hospital, Dublin 24, Ireland; Department of Health and Children, Hawkins House, Dublin 2, Ireland; Kildare and West Wicklow Doctors on Call, Naas, Co Kildare Ireland; TCD Department of Public Health and Primary Care, Tallaght Hospital, Dublin 24, Ireland

**Keywords:** GP Co-operative, Out of hours consulting, Intervention, Complaints, Risk reduction

## Abstract

**Background:**

General Practice Co-Operatives provide most out of hours care in communities in Ireland. Limited data exists on patient complaints. This study reports on complaints at Kildare and West Wicklow Doctors on Call (‘K Doc’), a GP Co-Operative in Ireland, examining the impact of a formal risk reduction strategy implemented (2010-2013). The aim of the study was to determine if it was possible to reduce the rate of written complaints per 1000 consultations through a formal approach encompassing evaluation of complaints, improved communication in relation to complaints, and more direct use of insights gained from complaints analysis in continuing professional development at the Co-Operative.

**Methods:**

Initially, complaints submitted over an 18 month period (01.06.08 to 31.12.09) were analysed. Complaint rate (number of complaints per 1000 consultations), complainant demographics, aspects of complaint response at the Co-Operative, and nature of complaint were recorded. Based on analysis, a risk reduction strategy was undertaken, including procedural change, focused training and education. Areas selected for improvement during a second phase of data collection included complaints rate, timeliness of Co-Operative response to complaint, and rate of complaint notification to patient’s GP. Further analysis was then carried out over a 45 month period (01.01.10 to 30.09.13).

**Results:**

From 2008-2013, 216,716 patient consultations occurred. Complaints were received from 131 individuals, regarding 125 patients. Following introduction of risk reduction strategy, complaints rate reduced by 36 %, from 0.77 to 0.49 per 1000 consultations (*p* = 0.02) between the two periods of data collection. Timeliness of response from Co-Operative to the complainant improved from 63 % to 75 %. Notification of complaint to the patient’s GP improved from 48 % to 96 %.

Most complaints were not associated with medically significant events. The largest categories of complaint related to clinical care (55 % *n* = 69), cost (46 %, *n* = 58), communication (42 %, *n* = 53), and process of care (15 %, *n* = 19). Mothers of affluent paediatric patients were most likely to make formal complaints.

**Conclusions:**

This study reports a statistically significant reduction in complaints rate of 36 % following introduction of risk reduction strategies at a GP Co –Operative. Out of hours consulting is known to be an area of high medical risk. Findings are of interest where number and costs of complaints against GPs are elsewhere reported to be rising, contributing to medical inflation, and to public concern.

## Background

There has been considerable change in out-of hours primary care in Ireland in the past 20 years. Prior to the establishment of the first GP Co-Operative by GPs at St. James’s Hospital Dublin in 1998, the average on‐call commitment of rural general practitioners was sixty six hours per week, and forty two hours per week for city colleagues [1]. The development of GP Co-Operatives has been an important and scaleable development in providing structured accessible out of hours care for patients, as well as managing the out of hours workload, and improving quality of life for GPs [2]. In Ireland, 14 out-of hours GP Co Operatives now exist. While individual Co-Operatives vary in terms of governance, structure and activity levels, overall levels of service provision are high, with almost 1 million contacts with Co Operative services in 2013 [3]. According to a national review of GP out of hours services published in 2010, 70 % of the population were then served by GP Co-Operatives, and patient satisfaction was rated at over 90 % for the majority of these services [4]. Despite high satisfaction ratings, complaints involving GPs in Ireland are rising, with claims more than doubling between 2007 and 2012 [5]. Further, highest settlements have occurred disproportionately in the out of hours setting, making it an environment of high medicolegal risk.

Commonly used measures to assess health care quality include data relating to patient survival and patient safety incidents. Studies indicate that data from analysis of complaints against individuals, departments and organisations also act as useful surrogate markers of healthcare quality [6, 7]. There are, to date, no published data relating to complaints made by patients in the out of hours setting in Ireland, either relating to the nature of the complaint itself, or to the process of handling complaints at the co-operative level. Further, there is a paucity of international data on complaints in this setting, with few studies, frequently making passing reference only to patient complaints in the GP Co Operative setting, and none readily identifiable which primarily describe complaints handling.

K Doc is an out of hours GP Regional Co-Operative, established in 2001 by GPs to provide urgent out of hours care in Kildare and West Wicklow. It now provides over 54,000 consultations per annum. It provides out of hours cover from 6 pm to 8 am weekdays and 24 h cover at weekends and bank holidays, including a minor injuries service [8].

This study examines the incidence, nature and handling of complaints at KDoc, identifies the number of complaints giving rise to medicolegal claims and adverse medical outcomes, and describes and evaluates the impact of a formal approach to medical risk reduction undertaken at the co-operative between 2008 and 2013, which involved a period of analysis followed by intervention, including the introduction of a formal risk reduction strategy during a second period of analysis. The two study periods study were determined empirically by the Co Operative Medical Committee, chiefly on the basis of allowing a meaningful volume of casehandling and experience to accrue.

## Methods

This is an analytic observational study, utilising a before and after design, set in a GP Out of Hours Co Operative. The study includes all written complaints from patients and/or carers submitted to K Doc during the periods 1st June 2008 to 31st September 2013. Verbal complaints and significant event reviews were excluded from the analysis. Data were collected from designated complaints folders containing written correspondence from complainants, all correspondence from the co-operative to the complainant, to any third party, consultation records, logs of telephone conversations, minutes of meetings of the medical committee of the Co-Operative and any meetings which took place between the complainant and Co-Operative representatives. Records were reviewed and data was extracted by two of three doctors (LV/RB/BOS) reviewing each case.

The first phase of data collection took place from 1st June 2008 to 31st December 2009.

The following information was recorded: patient demographics, source of complainant (patient themselves/relative/other), nature of complaint, location of patient contact (treatment centre/home visit/telephone consultation), background of attending GP (established GP/non established GP/GP Registrar), clinical outcomes, and action taken by the Co-Operative (timeliness of response, notification of complaint to patient’s own GP, changes to procedure). A note was made of complaints involving serious medical or legal outcomes. Throughout the study period, complaints were communicated to the Medical Director, upon receipt of which the complaint was communicated to the treating GP, requesting their formal written observations, following which a detailed response to the complainant was issued, and any identified changes to casehandling procedures were agreed by the Medical Committee and communicated to the Co Operative generally.

Timeliness of response is known to be important in terms of satisfactorily responding to patient complaints. Notification of the complaint to the patient’s own GP was felt to be important. From the perspective of clinical governance, care provided at the Co Operative is provided to the patient by arrangement with the patient’s own GP, who therefore has a professional responsibility for the care provided. Notification was felt to strengthen professional responsibility, and to render it more effective in the interest of the patient. Complaints frequently related to more than one aspect of care. For this reason we recorded each aspect of care which the individual complaint related to; thus the total number of aspects of care recorded exceeds number of complaints.

Following the findings of the first phase of data collection, the Co-Operative Medical Committee put in place a formal and organised risk reduction strategy (Table 3). The strategy included elements identified thorugh formal and informal consultation with GPs, and through reflection and analysis of complaints and significant incident reviews during the first phase of data collection. It included a range of procedural change, a commitment to improved selected key metrics, placing emphasis on improving care through continued systemmatic reflection on complaints, through provision of tailored and accredited continuing medical education activities to sub groups within the Co Operative (GP Trainees/Late Shift Doctors/GP Members/Co Operative Nursing Staff), and engagement with external educational resources, to improve the level of in house expertise in avoiding and handling complaints (particularly Medical Protection Society risk management seminars, and the State run Clinical Indemnity Scheme). Risk reduction activities were administered through the Co Operative Medical Committee and the Co Operative Medical Director. Part of risk reduction strategy included a formal decision to prospectively restrict GP membership of the Co Operative to GPs on the Specialist Register for General Practice at The Irish Medical Council. It also included procedural changes to issue more detailed guidance to GP Members at the Co Operative limiting the number of hours per week they worked on call at the Co Operative, and also on the need for periodic resuscitation skills training, recommending this at a two yearly interval, and annually for GPs working late shifts. Activities also included monthly written advisories from the Medical Director relating to changes in procedure, and periodic scheduling (quarterly) of accredited CPD activities for Co Operative GPs and Nursing Staff on topics directly suggested by reflection of complaints received, and in consultation with Co Operative staff, some of whom had been involved with a complaint.

Three key metrics were selected as targets for improvement for the second phase of the study (Table 1); the number of complaints per 1000 consultations, the timeliness of the co-operative response to the complaint, and the rate of complaint notification to the individual patient’s own general practitioner. Timeliness of the Co-Operative response was felt to be important, as delayed responses are recognised as an aggravating factor in handling complaints generally, whereas prompt and thorough responses are known to be valued by the complainant, and increase the likelihood of more effectively addressing the concerns raised by the complainant.

**Table 1:** Comparison of results between first and second phase

Criteria	Target	Phase I (*n* = 49 complaints)	Phase II (*n* = 76 complaints)
Reduction in complaints rate to 0.6 per 1,000 consultations	100 %	0.77 per 1,000 consultations	0.49 per 1,000 consultations
Written response to complainant within 2 weeks	90 %	63 %	75 %
Written notification of complaint to patient’s own GP	100 %	48 %	96 %

Rate of notification of the complaint to the patient’s own GP was felt to be important for two reasons. Firstly, given that the care provided was provided at the request of the patient’s GP, it is considered necessary to appraise the GP in instances of complaint. Secondly, given that the service is provided in the context of the Co Operative model, it was felt to be important for the patients, the Co Operative, and both the patients’ GP and the doctor providing care that the patient’s GP was notified, consolidating the shared responsibility between the GP and the Doctor on Call. As published data relating to complaints in the out of hours settings is limited, international standards were not identified in this regard, but were based on features considered to be part of good complaints procedure [9].

A second phase of data collection took place from 1st January 2010 to 31st September 2013. This time period was selected to ensure sufficient time elapsed in order to assess impact of the intervention. Comparison was made between complaint rate and identified metrics before and after the introduction of the risk reduction strategy.

With respect to statistical analysis, the difference observed between the complaints rate (described as the number of written complaints received per 1000 consulations) from the first phase of data collecton and the second phase of data collection were compared, and the rate ratio for the difference between the complaints rate between the two phases examined. Consultation rates were calculated from the electronic medical database used at the Co Operative (Adastra), where all patient contacts are registered by administrative staff at the point of contact of the patient with the service.

## Results

Over the total period studied, 216,716 patient consultations took place. Written complaints were received from 131 individuals, relating to the care of 125 patients.

A minority of complaints (30 %, *n* = 40) were made by the patient themselves, with most made by a family member on behalf of a patient (60 %, *n* = 78), the largest group of whom were mothers of minors, who comprised most complaints (60 %, *n* = 46) which were made by family members on behalf of a patient, and particularly those who were ineligible for medical care under the Primary Care Reimbursement Scheme (ie more affluent families). Complaints were sometimes made by non family members (10 %, *n* = 13). Of 125 patients whose care was the subject of a written complaint, most complaints related to the care of patients under 18 years of age (49 %, *n* = 61), whilst those over age 66 accounted for a small proportion (11 %, *n* =8). These proportions broadly reflected the age distribution of the whole population of patients attending, 49 % of which were aged 0 to 18, 40 % were aged >18 to 65, and 11 % were aged 66 or older.

Most complaints (83 %) related to consultations which had taken place in The Treatment Centre, with Treatment Centre consultations comprising 90.5 % of all consultations for the study period. The doctor at the centre of the complaint in most cases was an established GP (50 %, *n* = 64), with non established GPs accounting for 39 % of complaints (*n* = 11). GP Registrars (Trainee GPs in their third or fourth year of training) resulted in the lowest number of complaints overall (11 %, *n* = 14). For the purpose of this study, an established GP was considered to be a GP in practice in the catchment area of the Co Operative, as a principal or assistant, and on the specialist register of the Irish Medical Council for general practice.

Complaints were made regarding different aspects of care relating to 125 patients, summarised in Table 2.

**Table 2:** Content of Complaints

Concerns regarding clinical care	55 % *n* = 69
Cost	46 % *n* = 58
Communication	42 % *n* = 53
Process of care	15 % *n* = 19
Other	7 % *n* = 9

At the end of the first phase of data collection, 63,618 patient consultations had taken place with 49 complaints during this period (complaints rate 0.77 per 1,000 consultations).

Co-Operative responses to complaints were analysed; 63 % (*n* = 31) of complaints were responded to in writing to the complainant within two weeks. The patient’s own GP was notified of the fact that a complaint had been made in less than half of cases (48 %, *n* = 24).

The Co-Operative then engaged in a process of organised risk reduction (Table 3). Targets were set by the Co-Operative for the second phase in three key areas: the complaint rate per consultation, the timeliness of the Co-Operative response to the complaint, and the rate of notification to the individual patient’s own general practitioner. In addition to these targets, sustained and consistent communication at all levels within the Co Operative was undertaken with a view to improving care provided to patients in the light of analysis of previous and ongoing complaints.

**Table 3:** Co-Operative Risk Reduction Strategy

• Specialist General Practice Training became a condition of Co Operative membership
• All late “red eye” shift doctors required to attend quarterly CME meetings on selected topics/problem case reviews
• Regular Patient Satisfaction Surveys conducted and reflected back to Co-Operative members in detail
• Increased frequency of emergency skills training (BLS/AED)
• Risk Management Seminars conducted on site for members
• Improved induction training and support of GP Registrars
• GP Registrar appointed to Co-Operative Medical Committee
• Individual doctors, where felt appropriate by Medical Committee requested and required to attend Regional CME Tutor for CME

At the end of the second phase of data collection, the complaint rate reduced from 0.77 to 0.49 per 1,000 consultations. The rate ratio of 0.64 (95 % CI: 0.44 to 0.92) showed a significant reduction of 36 % following the intervention (p value = 0.02). All analyses were performed in statistical programme R (*A language and environment for statistical computing*. 2013 [cited 2015 10.12.15], available from: http://www.R-project.org/. We used prop.test function to test the difference in rate of complaints between two phases of data collection.

Timeliness of the response from the Co-Operative to the complainant improved from 63 to 75 %. Written notification of the complaint to the patient’s own GP improved from 48 to 96 % following the intervention.

In 90 % of complaints overall, there were no adverse medical outcomes. Two complaints were the subject of a Medical Council investigation, neither of which were upheld, and a third complaint resulted in the Co Operative engaging as a co complainant with the original complainant to The General Medical Council in the United Kingdom, to where the doctor involved in the complaint had moved. The registration of the doctor was subsequently endorsed. In the minority of cases where an adverse medical outcome was evident, the Co Operative engaged closely with the Complainant, and was seen to evidently modify casehandling/procedure, to actively feed back to Co Operative Team members involved in care, and in two instances to forward modest costs (< e 1500) directly to complainants wthout predjudice, where adjudged appropriate and necessary in the light of additional expenses and inconvenience to the complainants.

In no instance did complaints relating to care of patients result in civil litigation.

## Discussion

### Summary of main findings

A total of 63,618 consultations in the first period of data collection resulted in 49 formal complaints to the Co-Operative (complaints rate 0.77 per 1000). Following a period where risk reduction strategies were undertaken at the Co-Operative, the second period of data collection included 76 written complaints to the Co-Operative out of a total of 153,098 consultations. The complaint rate significantly reduced by 36 % from 0.77 to 0.49 per 1,000 consultations (p value = 0.02). A number of themes were identified, including complaints relating to concerns with quality of clinical care, cost, communication, and process of care at the Treatment Centre.

The largest group of complaints overall (55 %, *n* = 69) related to concerns regarding clinical care. In these instances the patient either disagreed with diagnosis, disagreed with choice of medication, failed to respond to medication prescribed, had a delayed diagnosis, or had to reattend their own GP for further follow up. This is consistent with previously published data on complaints in out of hours setting, where the largest proportion relate to complaints where either a delay or a failure in diagnosis or referral occurred, usually when the condition worsened, and required further medical attention [10].

It is important to interpret this in the context of out-of-hours clinical care, where the attending doctor may see a patient at an earlier stage in their illness, often with little knowledge of their past medical history, meaning that diagnoses will occasionally be delayed or, in hindsight, seemingly missed. Further, the absence of pre existing personal knowledge increases the risk of miss communication. These characteristics of out of hours clinical care highlight the importance of giving clear advice on follow, up should the patient not improve, documenting clear management plans as well as documenting all relevant clinical findings, both positive and negative at the time of the inital assessment. Ascertaining patient expectations and ensuring they are well grounded is also relevant in this context. During the second phase of this study, these observations were repeatedly communicated to Co Operative Members individually and in CPD organised by the Co Operative.

It was difficult to draw firm conclusions regarding data relating to background of the attending GP, as there was continual change in the composition of the Co Operative GP membership over several years, and during both phases of the study. In general, the proportion of duty carried out by non established GPs reduced during the entire study period. For the purposes of the study, non established GPs included Doctors who were working on the duty roster who were not GP Principals in practice in the catchment are of the Co Operative.

It was reassuring to note that despite a proporton of complaints relating to clinical care, in 90 % of cases it was considered by the Medical Committee at The Co Operative that there were no adverse medical outcomes. The study did not address issues relating to patient expectations, however previous research in the UK has shown that meeting or failing to meet idealised expectations of care is an important determinant of patient satisfaction in out of hours care [11], highlighting the importance of managing patient expectations, especially in an era of increased demands on out of hours care.

Whilst cost was a feature in 46 % of complaints (*n* = 58), it is interesting to note that concurrent patient satisfaction surveys (not yet published) during this period consistently rated the service as ‘good value’ for money overall (personal communication, Ken Bailey). Further, cost of consultation at the Co-Operative remained fixed over the period studied.

Communication difficulties were a factor in 42 % (*n* = 53) of complaints. These ranged from patients reporting difficulty seeking information, to perceived rudeness in the consultation, a perceived lack of understanding or concern, as well as poor explanation of illness and of prescription, in keeping with previous studies relating to complaints in general practice [12]. Poor communication is known to be an important factor in malpractice claims [13, 14]. In this study, the introduction of risk reduction strategies at the Co-Operative contributed to a statistically significant reduction in complaints of 36 % (from a rate of 0.77 to 0.49 complaints per 1000 consultations).

Most complaints were not made by the patient themselves, but by a family member on their behalf (59 %, *n* = 79). The largest category of complainant were mothers of minors (35 %). Analysis showed that those most likely to make a formal complaint were female (70 %), private patients (73 %) and the complaint related to the care of minors (45 %) aged 18 or younger, who comprised 40.6 % of all patients attending during the study period. During the study period (2008-2013), approximately half of all consultations at the Co Operative related to the care of fee paying patients, who were ineligible under the Primary Care Reimbursement Scheme (‘private patients’). In general terms, these are more affluent enfranchised individuals, and on the basis of this data, mothers of ‘private’ paediatric patients were most likely to make a formal complaint.

Previous studies found that observed differences in satisfaction between people of different groups may also be explained by idealised expectation mismatch [15, 16]. While this study examined patients’ dissatisfaction with out of hours care, it did not address issues related to patient expectations. This may be valuable, as patients with inappropriately high expectations may be dissatisfied with optimal care, while those with inappropriately low expectations may be satisfied with deficient care. In this regard, the experience of PCRS eligible (or ‘public patients’) is important.

### Strengths and Limitations of the Study

This study involved a complete analysis of all written complaints submitted to the Co-Operative over the period studied, and therefore avoided sampling error. The Co-Operative had a complaints system in place, ensuring relevant data was available. To the authors’ knowledge, this is the only study in the literature to specifically analyse complaints made in out of hours primary care in Ireland. However, findings relate to one co-operative only, and this reduces generalisability. Further, it is possible some complaints were unrecorded and further, not everyone who is dissatisfied makes a complaint. It is also possible that complaints relating to process of care at the Co-Operative may have been biased by a negative health outcome. As data was retrospectively collected some data may have been incorrectly recorded or classified, however this was addressed by corroboration of multiple information sources, and by the use of two clinicians reviewing each case. All data was handled in full compliance with the procedures of the Office of the Data Protection Commissioner, and no identifiable patient data was communicated outside the Co Operative.

## Conclusions

This study reports a statistically significant reduction in complaints rate following a process of sustained risk reduction at a GP Co –Operative. The measurement of patient experiences and satisfaction is an important component of evaluating out of hours services. In the UK, particular emphasis is placed on measuring patients’ satisfaction with the healthcare they receive as an important health outcome in itself [17]. This study demonstrated the importance of complaints analysis as a useful part of quality assurance, quality improvement, and the organisational benefits which arise when complaints are positively considered, analysed and systematically utilised as an opportunity to closely inform continuing professional development within a clinical service, and to monitor and improve services to patients over time.

## Abbreviations

AED, Automated External Defibrillator; BLS, Basic Life Support; CME, Continuing Medical Education; GP, General Practitioner; K Doc, Kildare and West Wicklow Doctors on Duty Ltd.
